# Supplementary material for: DNA methylation loss promotes immune evasion of tumours with high mutation and copy number load
Source: Nat Commun. 2019 Sep 19;10:4278. doi: 10.1038/s41467-019-12159-9 (PMC6753140; doi:10.1038/s41467-019-12159-9)
Supplement: Supplementary file 1 — Supplementary Information [file 41467_2019_12159_MOESM1_ESM.pdf]

Supplementary Information

DNA methylation loss promotes immune evasion of tumours with high mutation and copy number load

Jung et al.

Supplementary Figure 1

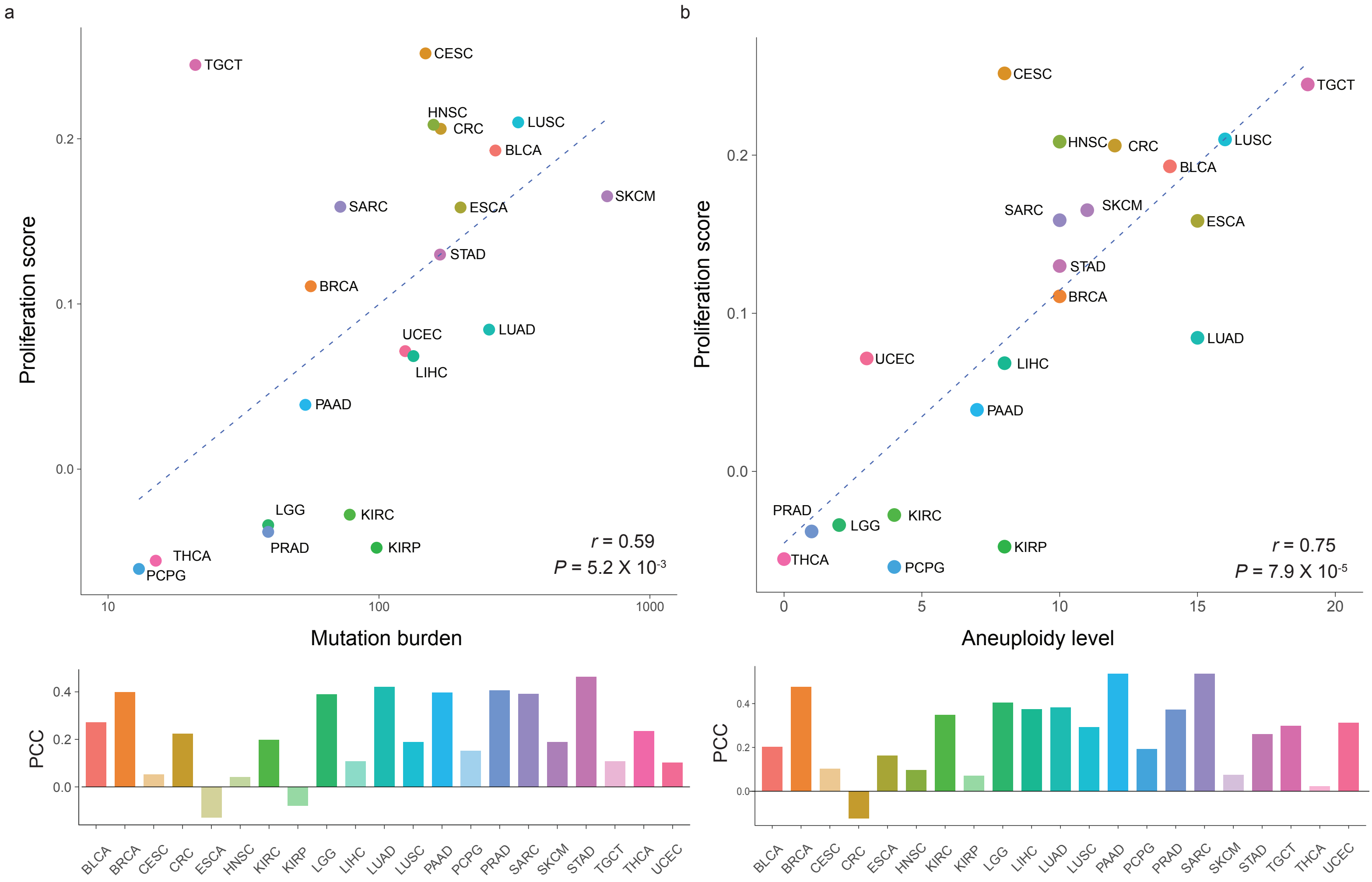

Correlation between cell proliferation markers and mutation burden (**A**) and aneuploidy level (**B**) across and within 21 cancer types. The median values were obtained for each cancer type, and statistical significance was evaluated using Spearman's correlation (upper scatterplots). For the correlation within each cancer type (lower bar graphs), Spearman's partial correlation was used to adjust for tumour purity. Tumour types showing significant partial correlation coefficient ($P < 0.05$) were shaded in darker colours.

Supplementary Figure 2

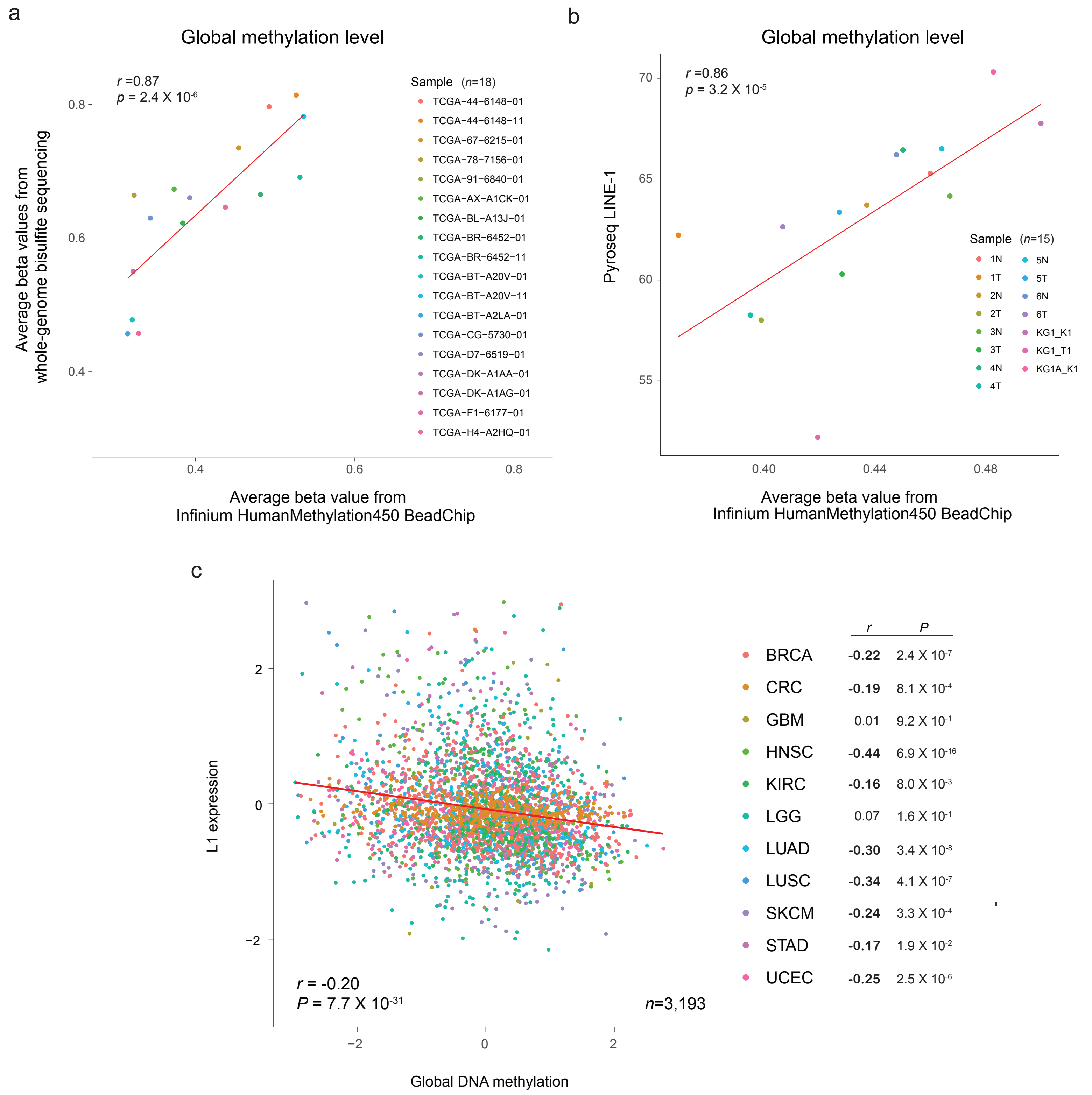

Correlation of global methylation levels measured by array versus sequencing. (**A**) We obtained the average beta value of evolutionarily young LINE-1 probes from the HumanMethylation450 BeadChip array for each of the TCGA samples for which whole-genome bisulfite sequencing data was available. The average beta value of all CpG sites that fell within evolutionarily young LINE-1 elements was obtained from the whole-genome bisulfite sequencing data for the same samples for comparison. (**B**) We obtained the average beta value of evolutionarily young LINE-1 probes from the HumanMethylation450 BeadChip array for each of samples for which LINE-1 pyrosequencing data were available (Nat. Biotechnol. 2016 Jul;34(7):726-37). (**C**) Correlation of the array-based global methylation levels and LINE-1 expression levels. The normalized LINE-1 expression levels were derived from RNA-seq data and compared to the global methylation levels measured by the LINE-1 probes. This analysis was confined to 11 tumour types. Significant ($P < 0.05$) correlations are highlighted in bold. (**A-C**) The Spearman correlation coefficient and its P values are indicated.

Supplementary Figure 3

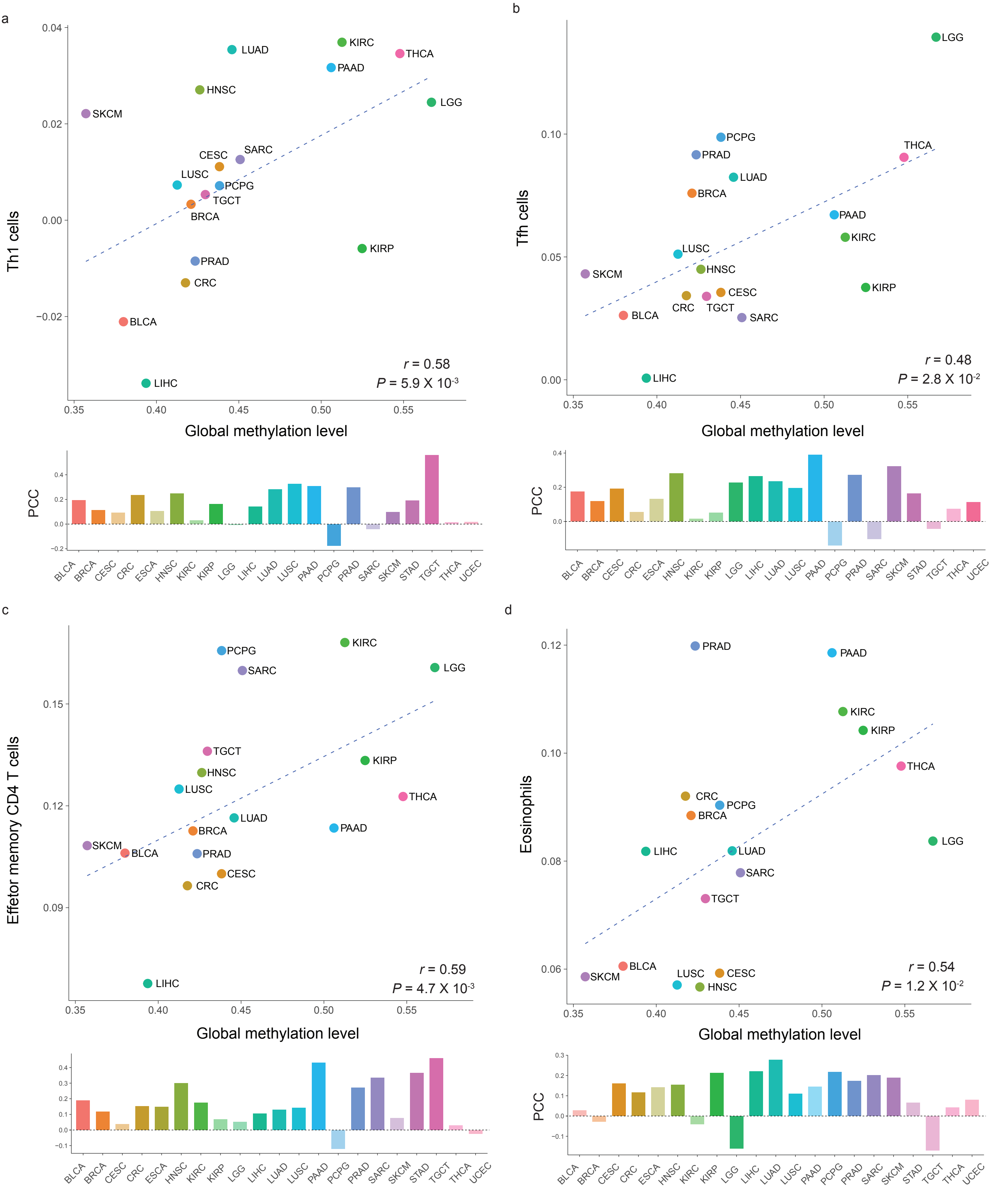

Correlation between genomic methylation levels and markers for Th1 cells (A), Tfh cells (B), effector memory CD4 T cells (C), and eosinophils (D). The median values were obtained for each cancer type, and statistical significance was evaluated using Spearman's correlation (upper scatterplots). Three outlier cancer types (ESCA, STAD, and UCEC) are not shown, but included when evaluating Spearman's correlation. For the correlation within each cancer type (lower bar graphs), Spearman's partial correlation was used to adjust for tumour purity. Tumour types showing significant partial correlation coefficient ($P < 0.05$) were shaded in darker colours. Markers for these immune cells were obtained from Thorsson et al. (Immunity. 2018 Apr 17;48(4)), and single-sample GSEA was utilized to measure the activity of the markers.

Supplementary Figure 4

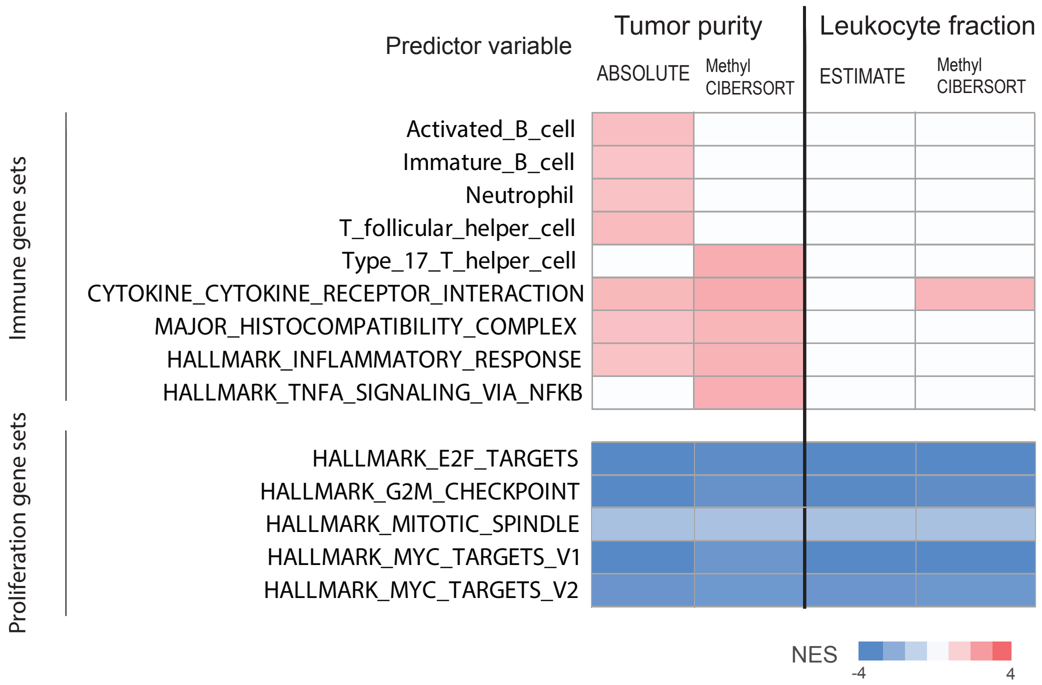

Tests for the effect of the leukocyte fraction. Heatmap of GSEA NESs for immune and proliferation gene sets. For each gene, a linear regression model was fit using mRNA expression level as response variable and global methylation, tumour purity or leukocyte fraction, age, and tumour type as predictors. GSEA was performed on genes with significant regression coefficients of the global methylation variable. Cells with significant NES are colour-scaled. Immune gene sets having negative NES values are not shown. This analysis is similar to what Taylor et al. (PMID: 29622463) did with aneuploidy level.

Supplementary Figure 5

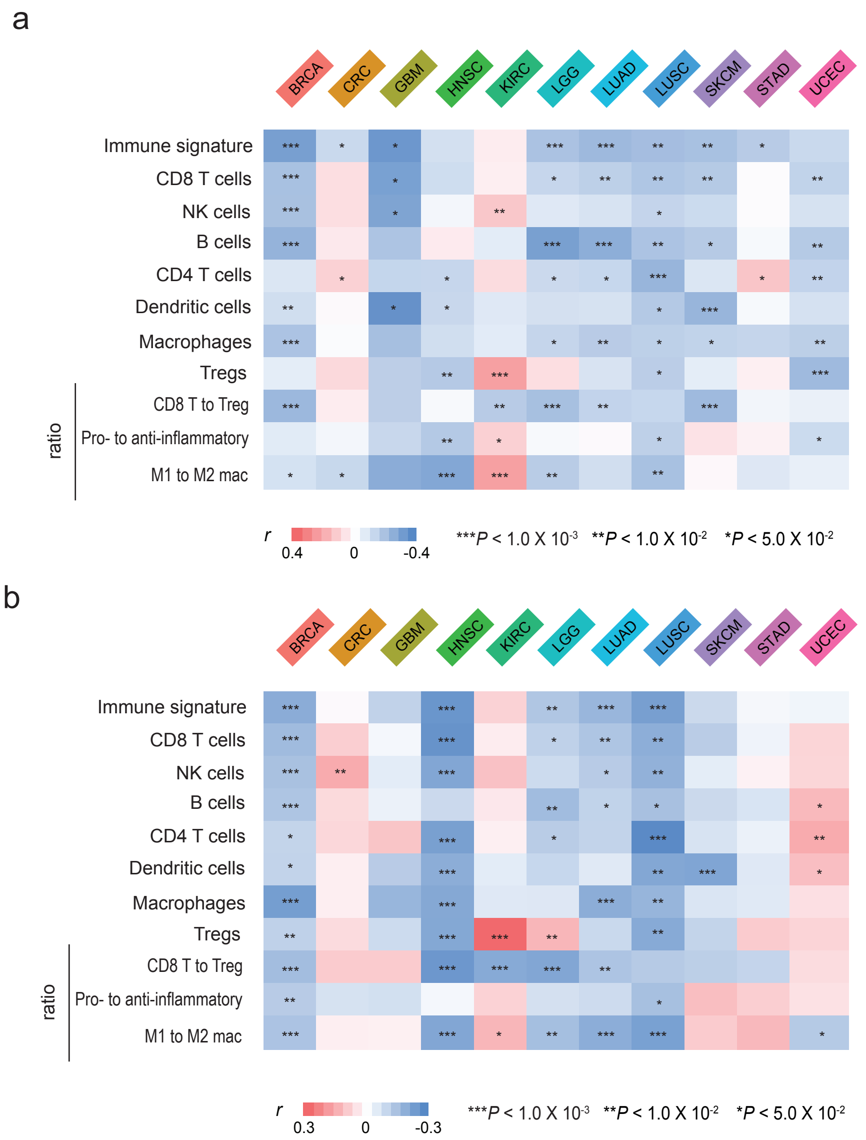

Correlation of (A) LINE-1 and (B) ERV expression with antitumour immune activity. CD8 T to Treg, Pro- to anti-inflammatory, and M1 to M2 mac indicate the ratio of cytotoxic T cell-specific genes to regulatory T cell-specific genes, the ratio of M1 (anti-tumourigenic) macrophage-specific genes to M2 (pro-tumourigenic) macrophage-specific genes, and the ratio of pro-inflammatory cytokine levels to anti-inflammatory cytokine levels, respectively. The gene sets used here were defined in Davoli et al. (Davoli et al., Science 355:261, 2017) for the 11 tumour types shown here.

Supplementary Figure 6

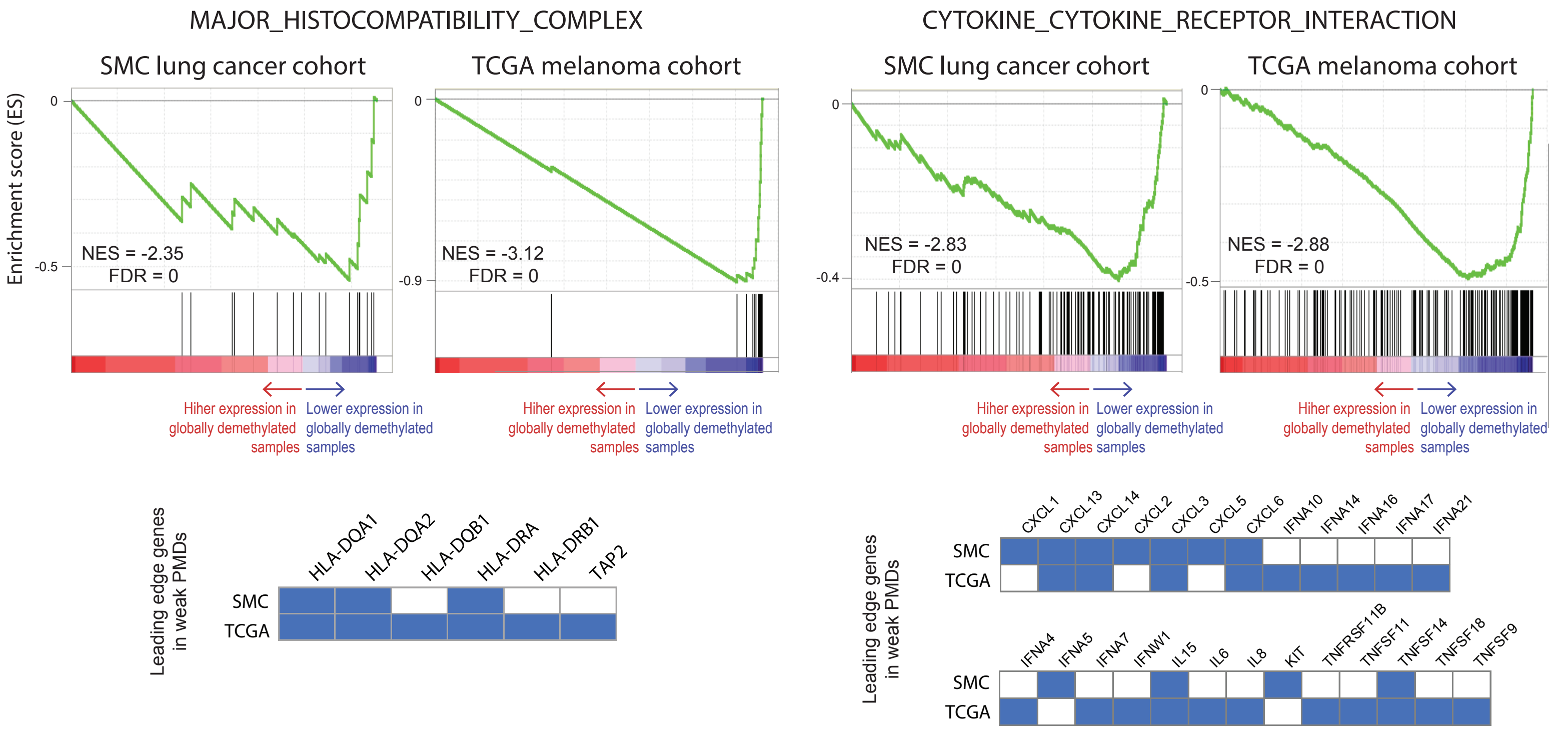

Significantly enriched pathways for genes repressed in globally demethylated tumours. Two representative immune pathways in the SMC and TCGA cohort are shown. For each tumour type, genes ranked by the t value obtained from comparing mRNA expression level between tumours with low and high global methylation levels were used for input into the 'preranked' module of the GSEA software with KEGG pathways and 'MAJOR HISTOCOMPATIBILITY COMPLEX'. The leading edge genes in the short PMDs are marked in blue.

Supplementary Figure 7

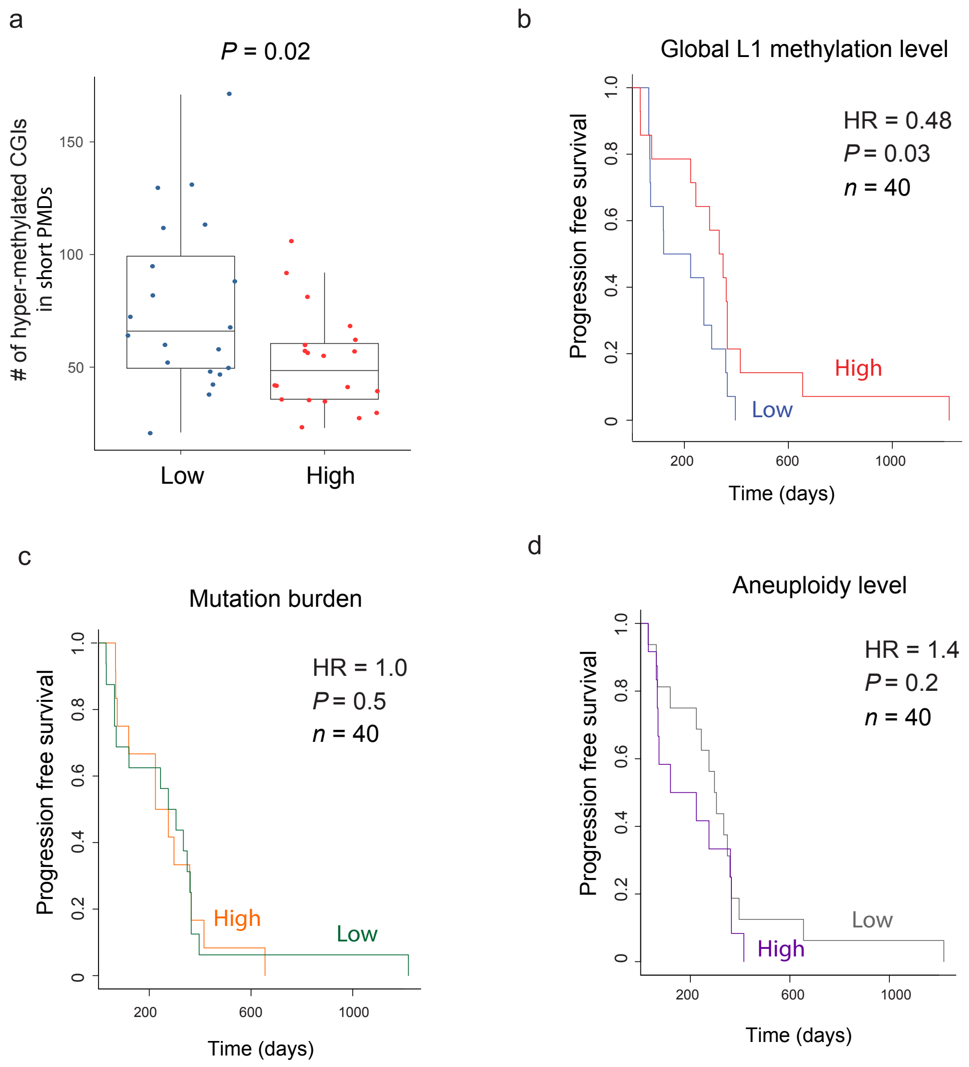

Effects of aberrant methylation on clinical responses in melanoma. (**A**) Comparison of the number of hyper-methylated promoter CGIs in the short PMDs between tumour samples with low and high global methylation levels. Two-sided Mann-Whitney U test was used to test the statistical significance. (**B-D**) Progression-free survival analysis. The one-sided flog rank test was used to compare survival curves estimated by the Kaplan-Meier method. Patients were stratified into two groups by the global methylation level (**B**), mutation burden (**C**), and the aneuploidy level (**D**).

Supplementary Figure 8

a

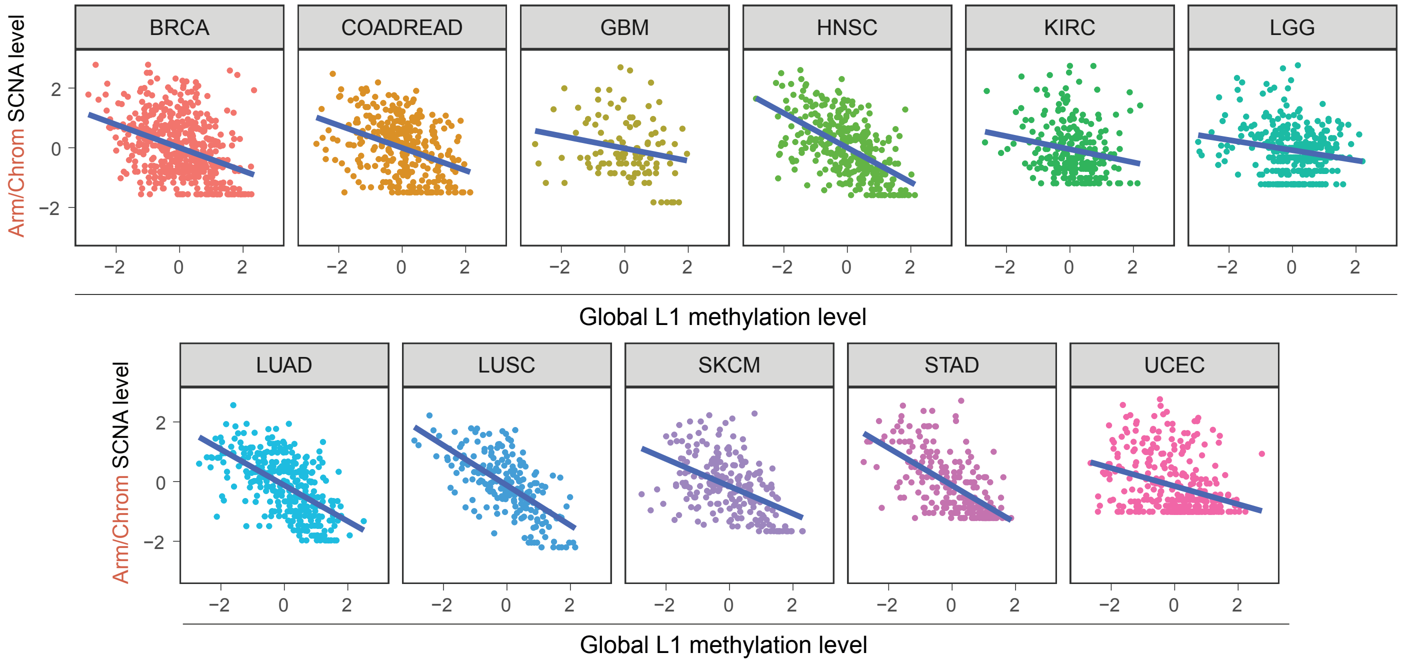

b

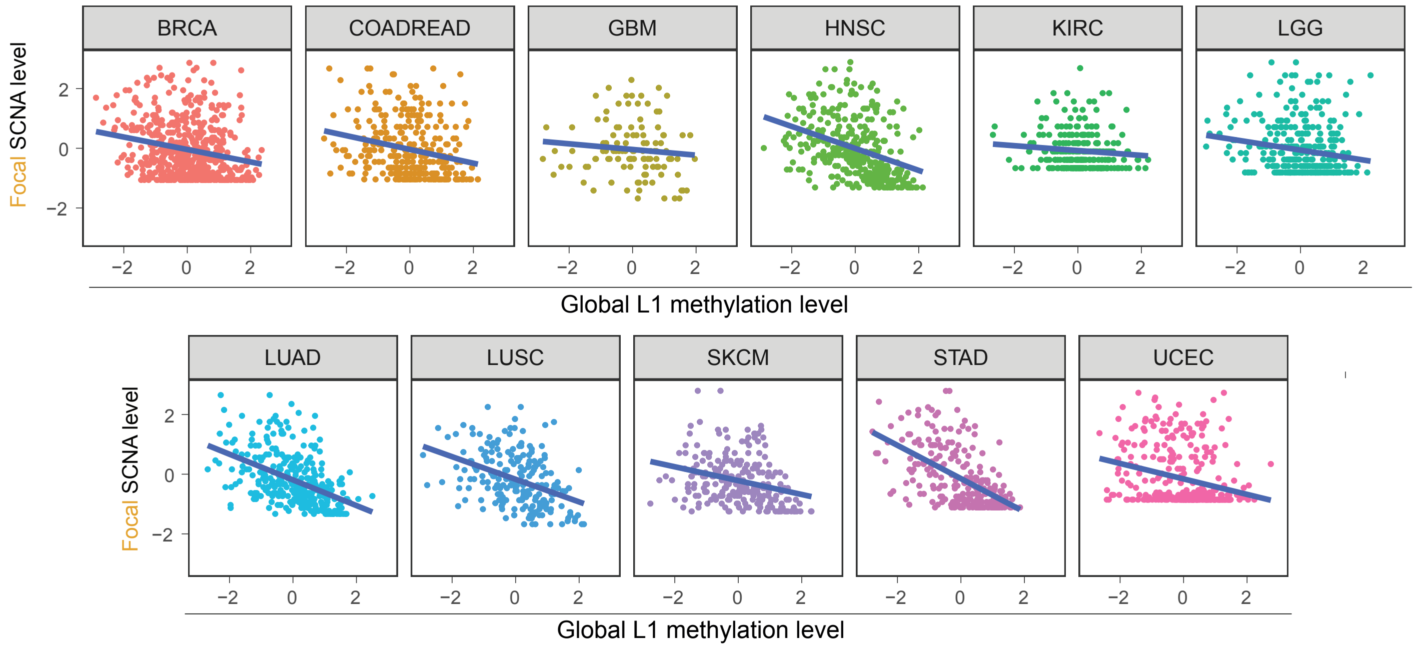

c

type	number	correlation analysis				regression analysis			
		am/chrom level		focal level		am/chrom level		focal level	
		r	p	r	p	beta	p	beta	p
BRCA	560	-0.37	1.62E-19	-0.20	3.13E-06	-8.99	3.73E-18	-4.55	6.59E-06
COADREAD	325	-0.38	2.35E-12	-0.26	1.70E-06	-7.38	1.32E-12	-3.98	8.56E-05
GBM	115	-0.23	1.28E-02	-0.14	1.30E-01	-2.89	4.59E-03	-1.04	2.99E-01
HNSC	326	-0.58	3.34E-31	-0.43	4.63E-16	-12.69	3.06E-30	-7.02	1.33E-11
KIRC	276	-0.23	1.54E-04	-0.13	3.73E-02	-5.32	2.13E-07	-8.81	1.43E-16
LGG	375	-0.20	9.14E-05	-0.23	8.26E-06	-5.18	3.70E-07	-4.36	1.71E-05
LUAD	334	-0.59	4.43E-33	-0.54	2.17E-26	-13.07	9.17E-32	-11.47	7.17E-26
LUSC	211	-0.66	2.06E-27	-0.43	8.18E-11	-12.60	1.93E-27	-6.43	8.47E-10
SKCM	231	-0.48	5.97E-15	-0.34	9.90E-08	-7.86	1.49E-13	-4.29	2.64E-05
STAD	244	-0.65	1.24E-30	-0.59	6.38E-24	-12.54	4.12E-28	-9.82	2.19E-19
UCEC	345	-0.32	1.64E-09	-0.31	5.92E-09	-5.79	1.63E-08	-5.13	4.79E-07

Correlation of global methylation levels with cSCNA and fSCNA levels. (**A**) Correlation of global methylation levels with cSCNA levels in each tumour type. (**B**) Correlation of global methylation levels with fSCNA levels in each tumour type. (**C**) The Spearman correlation coefficient and its P value (left), and regression beta coefficient and its P value (right), comparing the relationship of global methylation and cSCNA versus fSCNA in each tumour type.

Supplementary Table 1
Enriched pathways for late- (a) and early (b)-replicating genes

a. Enriched pathways for late-replicating genes

NAME	ES	NES	NOM p-val	FDR q-val	FWER p-val
TRAF6_MEDIATED_IRF7_ACTIVATION	-0.61	-2.31	0	0.004	0.002
CYTOKINE_CYTOKINE_RECEPTOR_INTERACTION	-0.4	-2.18	0	0.017	0.016
REGULATION_OF_IFNA_SIGNALING	-0.62	-2.17	0.009	0.013	0.019
JAK_STAT_SIGNALING_PATHWAY	-0.41	-2.11	0	0.015	0.029
RIG_I_MDA5_MEDIATED_INDUCTION_OF_IFN_ALPHA_BETA_PATHWAYS	-0.44	-2.04	0	0.025	0.056
ADP_SIGNALLING_THROUGH_P2RY12	-0.61	-1.9	0.004	0.073	0.185
AMINE_LIGAND_BINDING_RECEPTORS	-0.47	-1.84	0.005	0.101	0.287
REGULATION_OF_AUTOPHAGY	-0.48	-1.81	0.005	0.109	0.335
SIGNAL_AMPLIFICATION	-0.5	-1.8	0	0.105	0.359
SMOOTH_MUSCLE_CONTRACTION	-0.5	-1.78	0.024	0.118	0.439
ECM_RECEPTOR_INTERACTION	-0.36	-1.76	0	0.124	0.483
THROMBOXANE_SIGNALLING_THROUGH_TP_RECEPTOR	-0.53	-1.72	0.027	0.151	0.572
INHIBITION_OF_VOLTAGE_GATED_CA2_CHANNELS_VIA_GBETA_GAMMA_SUBUNITS	-0.49	-1.71	0.012	0.145	0.593
INTERFERON_ALPHA_BETA_SIGNALING	-0.39	-1.68	0	0.166	0.666
SIGNALING_BY_CONSTITUTIVELY_ACTIVE_EGFR	-0.52	-1.67	0.032	0.159	0.674
NOD_LIKE_RECEPTOR_SIGNALING_PATHWAY	-0.4	-1.67	0	0.15	0.675
ADP_SIGNALLING_THROUGH_P2RY1	-0.49	-1.66	0.016	0.153	0.708
INTEGRIN_CELL_SURFACE_INTERACTIONS	-0.35	-1.64	0	0.167	0.763
TRAF6_MEDIATED_NFKB_ACTIVATION	-0.51	-1.64	0.022	0.162	0.772
HEMATOPOIETIC_CELL_LINEAGE	-0.38	-1.63	0	0.159	0.777
RIG_I_LIKE_RECEPTOR_SIGNALING_PATHWAY	-0.37	-1.63	0.007	0.154	0.785
TAK1_ACTIVATES_NFKB_BY_PHOSPHORYLATION_AND_ACTIVATION_OF_IKKS_COMPLEX	-0.52	-1.62	0.022	0.158	0.812
G_PROTEIN_BETA_GAMMA_SIGNALLING	-0.47	-1.6	0.028	0.173	0.852
PRION_DISEASES	-0.44	-1.58	0.014	0.194	0.895
INWARDLY_RECTIFYING_K_CHANNELS	-0.42	-1.56	0.029	0.204	0.917
CYTOSOLIC_DNA_SENSING_PATHWAY	-0.37	-1.55	0.022	0.206	0.924
CLASS_A1_RHODOPSIN_LIKE_RECEPTORS	-0.28	-1.55	0	0.204	0.928
SIGNALING_BY_PDGF	-0.29	-1.52	0	0.233	0.96
COMPLEMENT_CASCADE	-0.42	-1.52	0.016	0.235	0.968
SMALL_CELL_LUNG_CANCER	-0.32	-1.51	0	0.243	0.976
REGULATION_OF_INSULIN_SECRETION_BY_GLUCAGON_LIKE_PEPTIDE1	-0.38	-1.5	0.044	0.237	0.976
AXON_GUIDANCE	-0.29	-1.5	0	0.23	0.976
NATURAL_KILLER_CELL_MEDIATED_CYTOTOXICITY	-0.3	-1.49	0	0.247	0.988

b. Enriched pathways for early-replicating genes

NAME	ES	NES	NOM p-val	FDR q-val	FWER p-val
SYSTEMIC_LUPUS_ERYTHEMATOSUS	0.7	2.58	0	0	0
RNA_POL_I_PROMOTER_OPENING	0.76	2.52	0	0	0
PACKAGING_OF_TELOMERE_ENDS	0.75	2.41	0	0	0
RNA_POL_I_TRANSCRIPTION	0.68	2.39	0	0	0
MEIOTIC_RECOMBINATION	0.68	2.38	0	0	0
AMYLOIDS	0.68	2.35	0	0	0
TELOMERE_MAINTENANCE	0.67	2.33	0	0	0
DEPOSITION_OF_NEW_CENPA_CONTAINING_NUCLEOSOMES_AT_THE_CENTROMERE	0.69	2.33	0	0	0
MEIOTIC_SYNAPSIS	0.65	2.28	0	0	0
MEIOSIS	0.61	2.24	0	0	0
RNA_POL_I_RNA_POL_III_AND_MITOCHONDRIAL_TRANSCRIPTION	0.59	2.19	0	0	0
CHROMOSOME_MAINTENANCE	0.57	2.1	0	0.00E+00	0.004
TRANSCRIPTION	0.51	1.95	0	0.003	0.047
IRON_UPTAKE_AND_TRANSPORT	0.55	1.71	0.004	0.083	0.781
MISMATCH_REPAIR	0.6	1.67	0.007	0.129	0.915
ASCORBATE_AND_ALDARATE_METABOLISM	0.59	1.66	0.005	0.127	0.924
GENERIC_TRANSCRIPTION_PATHWAY	0.41	1.64	0	0.156	0.965
CELL_CYCLE	0.41	1.64	0	0.151	0.967

Supplementary Table 2

Enriched pathways for genes in short- (A), intermediate (B), and long-PMDs (C).

A. Enriched pathways for genes in PMD-S

Pathway	FDR
OLFACTORY_TRANSDUCTION	1.50E-75
AUTOIMMUNE_THYROID_DISEASE	1.05E-13
ANTIGEN_PROCESSING_AND_PRESENTATION	2.07E-12
CYTOKINE_CYTOKINE_RECEPTOR_INTERACTION	3.02E-08
GRAFT_VERSUS_HOST_DISEASE	7.81E-08
REGULATION_OF_AUTOPHAGY	1.00E-07
NATURAL_KILLER_CELL_MEDIATED_CYTOTOXICITY	8.40E-06
INTESTINAL_IMMUNE_NETWORK_FOR_IGA_PRODUCTION	0.000159
ASTHMA	0.000172
RIG_I_LIKE_RECEPTOR_SIGNALING_PATHWAY	0.000603
CYTOSOLIC_DNA_SENSING_PATHWAY	0.000626
ALLOGRAFT_REJECTION	0.00068
JAK_STAT_SIGNALING_PATHWAY	0.001613
METABOLISM_OF_XENOBIOTICS_BY_CYTOCHROME_P450	0.003001
TYPE_I_DIABETES_MELLITUS	0.003001
RETINOL_METABOLISM	0.004821
TOLL_LIKE_RECEPTOR_SIGNALING_PATHWAY	0.004821

B. Enriched pathways for genes in PMD-I

Pathway	FDR
OLFACTORY_TRANSDUCTION	6.07E-22
NEUROACTIVE_LIGAND_RECEPTOR_INTERACTION	2.76E-14
ECM_RECEPTOR_INTERACTION	0.001616
CALCIUM_SIGNALING_PATHWAY	0.006112
LONG_TERM_DEPRESSION	0.0075

C. Enriched pathways for gene in PMD-L

Pathway	FDR
None	

Supplementary Table 3

Patient cohort for checkpoint blockade in lung cancer

Sample ID	Mutation burden	Global methylation level	Aneuploidy level	PFS	PD_Event.1_Censoring.0	Clinical benefit
3	176	0.462218282	9963319.531	28.1	1	DCB
20	93	0.497886714	0	1.133333333	1	NDB
258	53	0.414770954	141952144.3	3.966666667	1	NDB
325	217	0.357762176	373215792.9	3.866666667	0	na
378	117	0.414296576	102187138.2	13.73333333	0	DCB
488	353	0.434316952	535751134.6	1.6	1	NDB
541	180	0.362546225	349091677.2	1.2	1	NDB
573	366	0.410597593	114018200.9	1.5	1	NDB
618	141	0.349218209	263496773.3	1.366666667	1	NDB
658	117	0.403780292	141510150.7	1.066666667	1	NDB
678	489	0.396234214	191366708.2	22.43333333	0	DCB
700	68	0.428694023	17433991.68	1.366666667	1	NDB
720	325	0.294703592	307380376.1	4.066666667	1	NDB
756	603	0.280030877	658988768.2	1.5	1	NDB
825	194	0.251712972	784194239.2	1.433333333	1	NDB
830	223	0.479584284	0	2.933333333	1	NDB
947	401	0.387089023	58799436.69	20.6	0	DCB
990	43	0.325908608	285011126.5	3.166666667	1	NDB
1017	82	0.417538488	286678444.4	1.266666667	1	NDB
1066	42	0.395929718	48979857.76	0.766666667	1	NDB
1079	101	0.392672276	128159753.6	5.8	1	NDB
1104	1100	0.293149031	895819215	0.966666667	1	NDB
1145	178	0.421131362	5860926.472	2.433333333	1	NDB
1155	273	0.335438797	443235770.9	1.133333333	1	NDB
1164	175	0.293888515	520968472.1	0.366666667	1	NDB
1203	137	0.35971363	437223965.6	0.833333333	1	NDB
1208	242	0.349741738	927702954.8	0.933333333	1	NDB
1250	382	0.327435945	861780460	0.933333333	1	NDB
1297	427	0.416016984	68793176.27	10.5	1	DCB
1322	182	0.443427473	227632642	1.1	1	NDB
1327	441	0.42678156	99766614.32	6.833333333	0	DCB
1337	428	0.329661704	436129375.2	2.166666667	1	NDB
1352	1391	0.22054474	553574113.5	8.333333333	0	DCB
1358	328	0.377759517	649708156.1	8.566666667	1	DCB
1401	42	0.436672231	17494258.78	0.933333333	1	NDB
1412	202	0.264443355	545902993.4	0.1	1	NDB
1425	260	0.303500801	346192818.8	1.966666667	1	NDB
1443	314	0.270158864	836459992.2	1.766666667	1	NDB
1456	157	0.407939594	76492185.01	7.5	0	DCB
1490	169	0.315326936	403292217.7	0.9	1	NDB
1508	18	0.415487136	7542228.411	2.166666667	1	NDB
1510	273	0.377090295	465391056.4	10.8	0	DCB
1528	146	0.364790201	374128096.1	9.3	0	DCB
1554	38	0.400425652	5947504.686	1.233333333	1	NDB
1589	862	0.328377787	297587719.9	3.966666667	1	NDB
1619	13	0.455254226	7041421.973	2.733333333	1	NDB
1637	263	0.394335199	265842882.3	2.6	1	NDB
1708	266	0.450911862	2076055.089	5.6	1	DCB
1711	308	0.421240392	401532642.4	na	na	na
1751	327	0.390149399	225455169.6	4.833333333	1	NDB
1778	343	0.371084292	128523671.2	0.766666667	1	NDB
1809	156	0.324772186	400743320.7	1.466666667	1	NDB
1873	388	0.307758327	486476347.6	1.233333333	1	NDB
1883	245	0.413997537	199412472	4.366666667	1	DCB
1960	3424	0.413389522	349177478.5	1.166666667	1	NDB
2107	569	0.312709532	716225322	6.466666667	0	DCB
2126	285	0.415388193	145048834.8	2.1	1	NDB
2132	211	0.377043232	235992924.3	1.233333333	1	NDB
2133	524	0.381138966	93298957.65	3.766666667	1	NDB
2317	576	0.323139645	495812412.5	0.766666667	1	NDB

Supplementary Table 4

Summary statistics of the TCGA samples used

type	# of samples with molecular data and age information	# of samples with molecular data and age & stage information
BLCA	383	382
BRCA	625	618
CESC	284	0
CRC	351	335
ESCA	152	134
HNSC	497	424
KIRC	245	243
KIRP	257	235
LGG	490	0
LIHC	343	322
LUAD	360	356
LUSC	344	341
PAAD	136	134
PCPG	156	0
PRAD	372	0
SARC	208	0
SKCM	448	398
STAD	342	333
TGCT	128	79
THCA	453	451
UCEC	394	0
Total	6968	

Supplementary Table 5

Summary of samples with whole-genome bisulfite sequencing data

Sample	Global methylation level	Number of young LINE-1 elements used for estimation
TCGA-44-6148-01	0.796769211	70,851
TCGA-44-6148-11	0.814135612	71,120
TCGA-67-6215-01	0.734970609	71,054
TCGA-78-7156-01	0.663874472	70,050
TCGA-91-6840-01	0.50407867	70,186
TCGA-AX-A1CK-01	0.672933454	70,882
TCGA-BL-A13J-01	0.622092676	69,864
TCGA-BR-6452-01	0.664910171	71,944
TCGA-BR-6452-11	0.690754072	71,899
TCGA-BT-A20V-01	0.477100544	70,347
TCGA-BT-A20V-11	0.782246114	71,958
TCGA-BT-A2LA-01	0.456067806	67,732
TCGA-CG-5730-01	0.629759048	71,394
TCGA-D7-6519-01	0.660024395	71,005
TCGA-DK-A1AA-01	0.453509797	71,166
TCGA-DK-A1AG-01	0.549683454	71,239
TCGA-F1-6177-01	0.646124586	70,226
TCGA-H4-A2HQ-01	0.456630831	68,456
