# Supplementary material for: DNA methylation loss promotes immune evasion of tumours with high mutation and copy number load
Source: Nat Commun. 2019 Sep 19;10:4278. doi: 10.1038/s41467-019-12159-9 (PMC6753140; doi:10.1038/s41467-019-12159-9)
Supplement: Supplementary file 3 — Description of Additional Supplementary Files [file 41467_2019_12159_MOESM3_ESM.pdf]

File Name: Supplementary Data 1
Description : Estimated global methylation level for TCGA samples

File Name: Supplementary Data 2
Description : Genes with differential replication timing

File Name: Supplementary Data 3
Description : Differentially activated pathways between cohort samples with low and high global methylation level

File Name: Supplementary Data 4
Description : Genes specifically expressed in the immune system

File Name: Supplementary Data 5
Description : List of variants in cohort samples

File Name: Supplementary Data 6
Description : CNV segmentation data for cohort samples
